# Retroperitoneal cystic mature teratoma in an adult male: A clinical report of one case and review of the literature

**DOI:** 10.1097/MD.0000000000041284

**Published:** 2025-01-17

**Authors:** Zekai Zhang, Ling Zhang, Hua Zhang, Xiaoguang Huo, Wenzhe Xu, Yongting Zhang

**Affiliations:** a Department of Ultrasound, Zibo Central Hospital, Zibo, China; b Department of Anaesthesia, Qingzhou City People’s Hospital, Qingzhou, China; c Department of Respiratory, Zibo Central Hospital, Zibo, China.

**Keywords:** adult, cystic maturation, male, retroperitoneal, teratoma, ultrasound

## Abstract

**Rationale:**

A case of retroperitoneal cystic mature teratoma in an adult male. Retroperitoneal cystic mature teratoma is a type of teratoma. The disease has occult onset, does not have the typical characteristics of teratoma, and is difficult to distinguish from cystadenoma and other diseases. Cystic mature teratoma is benign, but it has a certain risk of malignant transformation. The purpose of this case report is to highlight the specificity of the case, collect and analyze the imaging features of the disease, and provide assistance for daily clinical diagnosis.

**Patient concerns:**

Adult extraglandular abdominopelvic teratoma is extremely rare, especially in men. These masses are most commonly found by chance and require surgical resection for diagnostic confirmation after a thorough examination.

**Diagnoses:**

We report a case of retroperitoneal cystic mature teratoma in an 86-year-old man. Abdominal ultrasound revealed a cystic mass in the abdominal cavity (possibly from the pancreas). Enhanced abdominal computed tomography showed a space-occupying lesion with a lack of blood supply in the hepatogastric space (above the pancreas), and the local boundary with the pancreas was unclear. The tumor was considered first, but pancreatic cystadenoma was not excluded.

**Interventions:**

Surgical resection is the most important treatment for retroperitoneal cystic teratoma, and the best adjuvant treatment needs further study.

**Outcomes:**

Postoperative pathological findings showed (retroperitoneal) mature cystic teratoma with lymphoid hyperplasia, lymphoid follicle formation, and mild to moderate atypical hyperplasia of squamous epithelium.

**Lessons:**

In conclusion, adult retroperitoneal cystic mature teratoma is a rare disease. This disease should be taken into account when we find a retroperitoneal cystic mass on our routine imaging. The shortcomings of this case report are mainly single case, small sample size, need to collect more cases, and long-term follow-up to summarize the characteristics of retroperitoneal cystic mature teratoma, in addition, this case did not undergo magnetic resonance imaging examination, magnetic resonance images cannot be analyzed.

## 1. Introduction

Retroperitoneal cystic mature teratoma is a type of teratoma which is rare in male extragonadal organs. Primary retroperitoneal teratomas account for 1% to 11% of all primary retroperitoneal tumors and are very rare, with only 10% to 20% of such tumors occurring in adults over 30 years of age. Women are twice as likely as men to develop the disease. Retroperitoneal teratoma lacks characteristic clinical manifestations in early stage. The diagnosis of this disease mainly depends on imaging examination and pathological diagnosis. Ultrasonography is a simple and easy operation, which is helpful to the localization and qualitative diagnosis of the disease, but it is difficult to distinguish from other cystic tumors. The final diagnosis often depends on the postoperative pathology.

We report a case that was first detected by ultrasound, but so far, ultrasound reports are rare.

## 2. Case report

This study has been reviewed and approved by the Ethics Committee of Zibo Central Hospital with the ethics number 2024 Yan No. 082.

**Figure 1.**
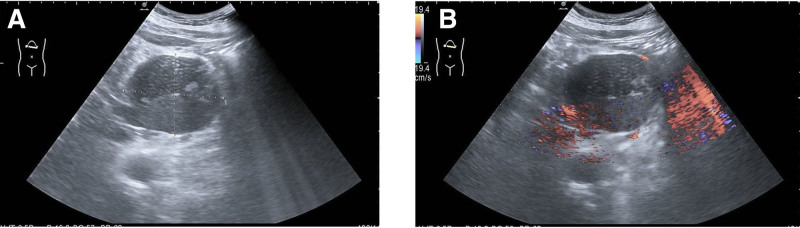
US image description. (A) Cystic mass between lower edge of left liver and upper edge of pancreas body, about 86 × 69 mm in size, clear boundary, close relationship with pancreas, poor boundary, and poor internal acoustic transmission. (B) Tumor CDFI: blood flow signals were seen in the wall of the sac. CDFI = color doppler flow imaging, US = ultrasonography.

In May 2022, an 86-year-old male patient underwent hepatobiliary, pancreatic, and splenic ultrasound examination in the western district of our hospital. Cystic mass was found between the lower edge of the liver and the upper edge of the pancreatic body, with a size of about 8.6 × 6.9 cm, clear boundary, close relationship with pancreatic body, unclear boundary, poor internal sound transmission, and blood flow signals were seen in some cystic walls. Ultrasonography of the liver, gallbladder, pancreas, and spleen showed: cystic mass in the abdominal cavity (probably originated from pancreas) (Fig. 1).

**Figure 2.**
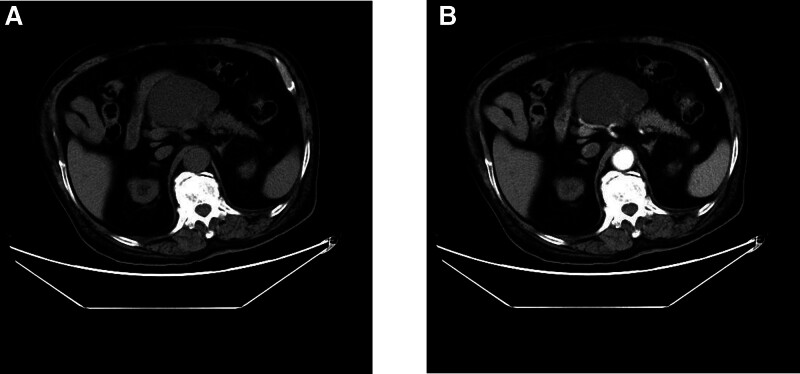
CT image description. (A) In the hepatogastric space area (above pancreas), circular mixed density shadow was seen, with a large range of about 80 × 83 mm, clear boundary, slightly irregular shape, uneven internal density, and compartmentalized and stratified changes. The CT value of the upper part of the lesion was about 20 HU and the CT value of the lower part was about 35 HU on plain scan. (B) Enhanced CT scan showed that the lesion was compartmentalized and enhanced, and the boundary between some enhanced components and pancreatic parenchyma was not clear. CT = computed tomography, HU = hounsfield unit.

In order to better understand the nature of the lesion, we carried out computed tomography (CT) enhancement examination for the disease number, and the enhanced CT showed a round-like mixed density shadow with a large area of about 8.0 × 8.3 × 10.0 cm was seen in the hepatogastric space area (above pancreas), with clear boundary, slightly irregular shape and uneven internal density, and separation and layered changes were seen. The CT value of the upper part of the lesion on a plain scan was about 20 HU, and the CT value of the lower part was about 35 HU. After an enhanced scan, the lesion was separated and enhanced, and the boundary between some enhanced components and pancreatic parenchyma was not clear. No abnormal density lesion is found in liver, no obvious abnormal enhancement lesion is found in the enhanced scan, and there is no dilatation of intrahepatic and extrahepatic bile ducts. The gallbladder is not big, the wall is not thick, and no abnormal density lesions are found. The spleen is not big and is homogeneous. No enlarged lymph nodes were found in retroperitoneum. CT showed: space-occupying lesion lacking blood supply in the hepatogastric space (above pancreas), with unclear boundary between the local area and pancreas. Tumor was considered first, but pancreatic cystadenoma was not excluded (Fig. 2).

Laboratory examination showed tumor marker screening (male 8 items): carbohydrate antigen 19-9 212.00 U/mL, neuron-specific enolase 19.00 ng/mL; biochemical routine + amylase: albumin 30.6 g/L, albumin/globulin ratio 1.0, total cholesterol 2.55 mmol/L, high density lipoprotein cholesterol 0.66 mmol/L, cystatin C 3.83 mg/L, low density lipoprotein cholesterol 1.64 mmol/L, prealbumin 92.0 mg/L, total protein 60.4 g/L, uric acid 493.0 µmol/L, small and dense low density lipoprotein 0.22 mmol/L, and complement C1q 143.30 mg/L.

**Figure 3.**
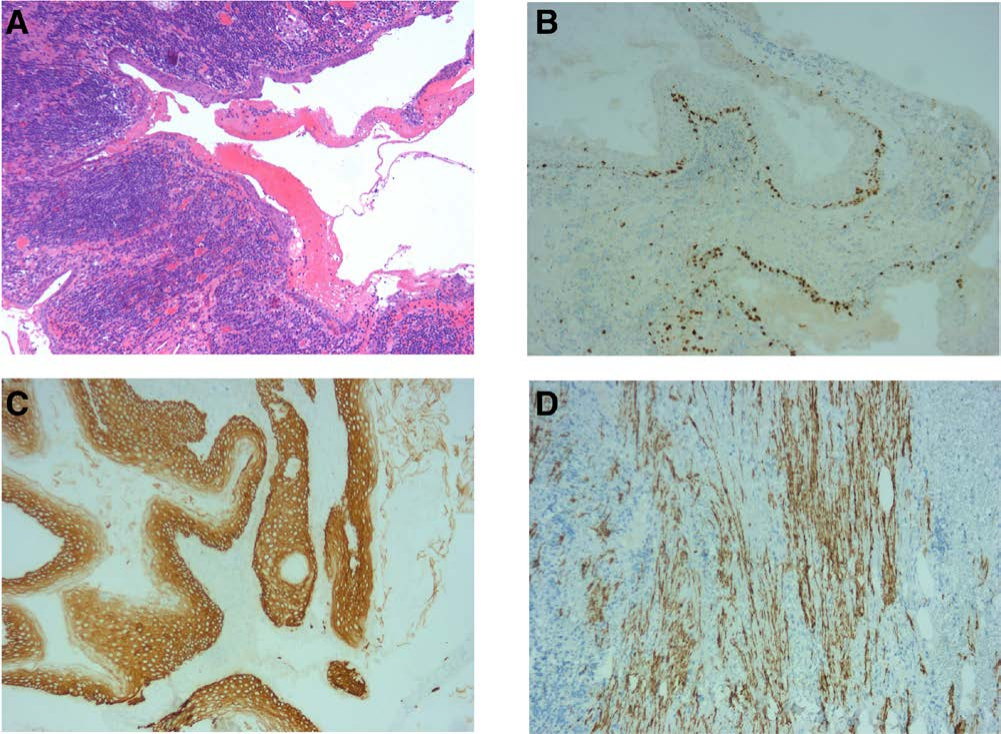
Pathological and immunohistochemical result description. (A) Cystic mature teratoma lymphoid hyperplasia. (B) Immunohistochemical result of cystic mature teratoma B (100×): ki-67+. (C) Immunohistochemical result of cystic mature teratoma C (100×): Desmin+. (D) Immunohistochemical result of cystic mature teratoma D (100×): CK56+.

Finally, laparoscopic retroperitoneal tumor resection was converted to open surgery under general anesthesia. Intraoperatively, the tumor was located retroperitoneally, with a size of about 9 × 9 × 7 cm. Postoperative pathology revealed (retroperitoneal) mature cystic teratoma with lymphoid hyperplasia, lymphoid follicle formation, and mild to moderate atypical hyperplasia of partial squamous epithelium (tumor size: 9 × 6 × 2 cm). Immunohistochemistry: vimentin: (+); desmin: smooth muscle (+); CKAE1/AE3, CK56: epithelium (+); CK8/18, CK7, P40: partial (+); 0CT-2: scattered (+); localized expression: CD20, CD3, CD79a, CD5, bc1-2, bc1-6, CD10: (+); Mum1, CD68: diffuse (+); c-myc, CD30: focal (+); TTF-1Myogenin, MyoD1, S-100, Olig-2, GFAP, SALL-4, ALK: (‐); Ki-67: (+) S accounted for 15% (Fig. 3).

## 3. Discussion

Retroperitoneal cystic mature teratoma is a type of germ cell tumor. They are histologically defined as comprising tissue derived from all 3 germ cell layers: ectoderm, mesoderm (most teratomas contain fat, an imaging marker, which is a derivative of mesoderm), and endoderm.^[1]^ Germ cells develop during embryogenesis and usually descend along a midline path to the pelvis to form ovarian cells or to the scrotum to form testicular cells. If these cells are unable to migrate along the urogenital ridge, the germ cells may deposit in extragonadal sites and be at risk of neoplastic transformation. Thus, teratomas can occur in the ovary, testis, anterior mediastinum, retroperitoneum, and skull in decreasing order of frequency.^[2]^ Teratomas are classified according to their histopathological findings into 2 types: mature and immature, containing adult and embryonic tissue, respectively. Microscopically, mature cystic teratomas are usually unicellular cysts containing tissue from all 3 germ cells, and may contain tooth, bone, and nerve tissue, making it easy to make an accurate diagnosis of teratomas in imaging.^[3]^ Primary retroperitoneal cystic mature teratoma is rare, with only a few cases reported in the medical literature and most commonly in children aged 1 to 3 years.^[4,5]^ In adults, the disease is rarer in men. Adult retroperitoneal cystic mature teratoma is usually present on the left side, near the upper pole of the left kidney, often presenting as a space-occupying lesion in the left adrenal gland.^[2]^ This is a rare case of retroperitoneal location. These lesions usually remain asymptomatic until they become large so as to produce symptoms of compression or mass effect that they are not detected.^[2]^ Retroperitoneal cystic mature teratoma lacks characteristic clinical manifestations in early stage. The diagnosis of this disease mainly depends on imaging examination and pathological diagnosis.^[3]^ Ultrasonography is the first choice of imaging examination for the diagnosis of teratoma, which is simple, safe, and low in cost. Most of them are round mixed echo masses in the retroperitoneal area with clear boundary. The echo inside the tumor is uneven, with strong light clusters and bright light spots, some accompanied by acoustic shadows and irregular hypoechoic dark areas. There may be septa inside the tumor, and there is no obvious blood flow signal in most of them. In our case, the ultrasound revealed a cystic mass between the inferior border of the left liver and the superior border of the pancreatic body, with clear borders, poor endosonicity and no obvious blood flow signal. There is no characteristic manifestation of teratoma in ultrasonic images, so it is difficult to diagnose teratoma. Because of its anatomical location, it is often difficult to distinguish from pancreatic cystadenoma and pancreatic pseudocyst.^[4]^ CT is highly sensitive and characteristic for the detection of teratomas, which often present as masses of mixed density with fat and calcification.^[6]^ In our case, enhanced CT of the abdomen showed a round-like mixed density in the hepatogastric space (above the pancreas), with septate and stratified changes. The enhanced scan showed lesion separation enhancement; no abnormal density lesion was found in liver, and no obvious abnormal enhancement was found in the enhanced scan; no enlarged lymph node was found in retroperitoneum. Because there was no obvious fat and calcification in this case, it was considered to be a cystadenoma.

CT and magnetic resonance imaging are of great significance in evaluating the anatomical relationship between the tumor and surrounding organs, whether there is distant metastasis, and clinical staging. The size and position of that tumor and the presence or absence of metastasis of adjacent organ provide great help for the later treatment.^[7]^ Ultrasound-guided or CT-guided biopsy is of great help in determining the nature and source of various abdominal organs. However, for retroperitoneal lesions, the difficulty of puncture is greatly increased because of the obstruction of anterior organs. For cystic lesions, it is often difficult to determine the nature of the lesion by means of a needle biopsy because the sampling is often unsatisfactory.

Laboratory examination of mature retroperitoneal teratoma is mostly negative, and some immature retroperitoneal teratoma may be accompanied by the elevated levels of alpha-fetoprotein, human chorionic gonadotropin, and carcinoembryonic antigen, which have certain guiding significance for the diagnosis and prognosis.^[2,8]^ Most mature cystic teratomas are benign, but malignant transformation of benign teratomas into various related tumors has been reported, with malignant transformation reported in about 0.5% to 3%, especially with increasing age. The most common type of cancer is squamous cell carcinoma. Other less frequent malignant changes include mucinous carcinoma.^[9]^ Therefore, surgical resection is often the best treatment for mature retroperitoneal teratoma. The prognosis of surgery is often better in adolescents than in adults.

## 4. Conclusion

In conclusion, adult retroperitoneal cystic mature teratoma is a rare disease. Because of its insidious onset and lack of corresponding specific imaging findings, it is difficult to distinguish from cystic mass lesions such as cystadenoma, and it is difficult to diagnose with many retroperitoneal cystic masses on imaging. Through the study of this case and review of related literature, we hope to help clinicians have a deeper understanding of retroperitoneal cystic mature teratoma and provide reference for preoperative evaluation.

## Author contributions

**Investigation:** Hua Zhang.

**Methodology:** Wenzhe Xu.

**Resources:** Ling Zhang, Xiaoguang Huo.

**Supervision:** Yongting Zhang.

**Writing – original draft:** Zekai Zhang.

**Writing—review & editing:** Yongting Zhang.
